# A circumflex coronary artery-to-right atrial fistula in a 10-month-old child

**DOI:** 10.5830/CVJA-2016-044

**Published:** 2016

**Authors:** Emrah Şişli, Mehmet Fatih Ayık, Muhammet Akyüz, Münevver Dereli, Yüksel Atay

**Affiliations:** Section of Paediatric Cardiovascular Surgery, Department of Cardiovascular Surgery, Faculty of Medicine, Ege University, Izmir, Turkey; Section of Paediatric Cardiovascular Surgery, Department of Cardiovascular Surgery, Faculty of Medicine, Ege University, Izmir, Turkey; Section of Paediatric Cardiovascular Surgery, Department of Cardiovascular Surgery, Faculty of Medicine, Ege University, Izmir, Turkey; Section of Paediatric Cardiovascular Surgery, Department of Cardiovascular Surgery, Faculty of Medicine, Ege University, Izmir, Turkey; Section of Paediatric Cardiovascular Surgery, Department of Cardiovascular Surgery, Faculty of Medicine, Ege University, Izmir, Turkey

**Keywords:** heart defects, congenital, atrial septal defect, vascular fistula, cardiac surgical procedures

## Abstract

A coronary fistula (CF) is a rare congenital cardiac anomaly in which there is a connection between the coronary artery and a cardiac chamber or a great vessel. In the paediatric population, a CF is usually asymptomatic. While the circumflex coronary artery (Cx) is the least common source of a CF, the right heart chambers are the most common location of drainage. Herein, we present a symptomatic 10-month-old boy with an atrial septal defect (ASD) in whom we incidentally detected a CF, which stemmed from the Cx and drained to the right atrium. Because the patient was symptomatic and his small size was not appropriate for percutaneous closure of the ASD, surgical closure of the ASD and CF was performed.

## Abstract

A coronary fistula (CF) is a rare congenital cardiac anomaly in which there is a connection between one or more coronary arteries and a cardiac chamber or great vessel.^1^-^3^ Herein, we present a paediatric case with a CF between the circumflex coronary artery (Cx) and the right atrium (RA).

## Case Reprot

In the follow up of a 10-month-old boy (weight 8 kg and height 70 cm) with a prenatal diagnosis of atrial septal defect (ASD), apart from the fixed splitting of the second heart sound, a prominent increase in the severity of the mid-systolic murmur at the pulmonary auscultation area was incidentally detected. On echocardiography, a new continuous jet flow into the RA indicative of a CF was detected.

**Fig. 1.**
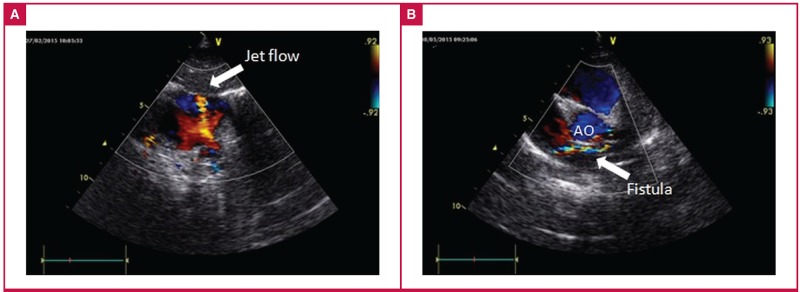
Pre-operative subcostal (A) and modified parasternal short-axis (B) echocardiographic views demonstrating the jet flow and trajectory of the coronary fistula.

His history showed he had had recurrent upper respiratory infection and failure to thrive so that both the weight and height of the patient were within the third and 10th percentiles. There was no evidence of myocardial ischaemia on electrocardiography. On echocardiography, the ASD (5 mm) was secundum type. The opening of the jet was sited adjacent to the superior cavo-atrial junction (Fig. 1A). Posterior to the aorta, turbulent flow of the CF originating from the left coronary system was detected (Fig. 1B). The Qp/Qs was 1.7.

**Fig. 2.**
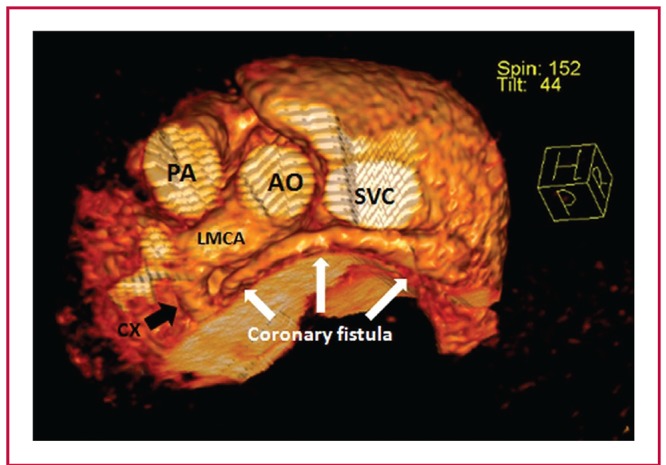
Three-dimensional reconstructed computed tomographic view demonstrating the trajectory of the coronary fistula. Note the dilated left main and circumflex coronary arteries. AO: aorta, LMCA: left main coronary artery, PA: pulmonary artery, SVC: superior vena cava.

In contrast-enhanced computed tomography, the left main and circumflex coronary arteries were dilated (6 and 4.5 mm, respectively). The CF originated from the proximal Cx and coursed posterior to the aorta before draining into the RA (Fig. 2). Because of the small size of the patient, percutaneous closure of both the ASD and CF was not appropriate therefore surgical closure was planned

After a median sternotomy and pericardiotomy, the CF was located adjacent to the posterior part of the superior cavo-atrial junction. Under cardiopulmonary bypass (CPB), the aorta was cross-clamped and cardiac electromechanical quiescence was established. Through a right atriotomy, the secundum ASD was primarily closed.

**Fig. 3.**
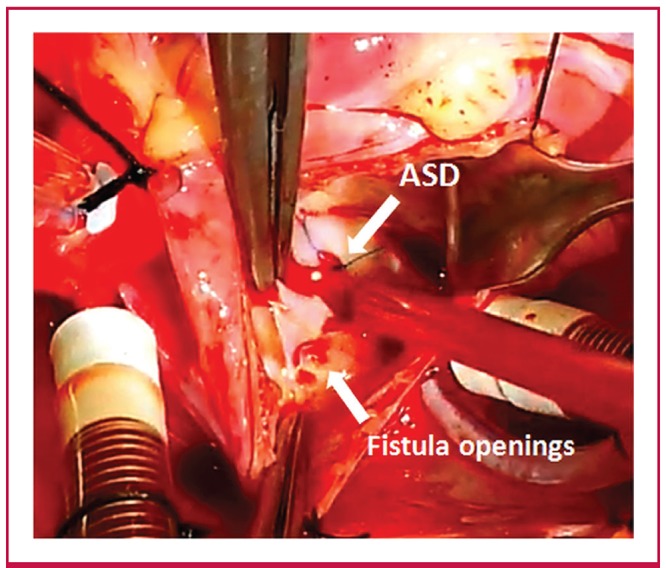
Surgeon’s view revealing the multiple openings of the coronary fistula. ASD: atrial septal defect.

It was detected under cardioplegic wash-out that the opening of the CF was located inferior to the cavo-atrial junction and it had multiple openings, which were connected with a loose membrane (Fig. 3). Following the primary closure of the openings from within the RA, the connection was checked with a second cardioplegic wash-out. Because of the loose membranous connection between the openings and to ensure that the fistulous connection was separated, the fistula was ligated outwardly, close to the opening of the RA. After weaning from CPB, no electrocardiographic changes indicative of myocardial ischaemia occurred.

The postoperative course was uneventful. In postoperative echocardiography, the atrial septum was intact and no turbulent flow in the right atrium was detected. Aspirin was given for three months. At the six-month follow-up visit, he was found to have gained weight (12 kg). Additionally, no turbulent flow within the atrium or posterior to the aorta was detected in echocardiographic evaluation.

## Discussion

As the use of selective coronary angiography became widespread, recognition of a CF has been improving since the 1950s.^1^ Sercelik et al. found the incidence of congenital CFs in the Turkish population was 0.08%.^4^ Among 286 cases with CF, the source of the CF was the right coronary artery in 56% and the left coronary system in 36% of cases.

In the literature, while the Cx was the least common source of a CF, the right heart chambers were the most common location of drainage.^1^-^7^ Although spontaneous closure has been demonstrated, either surgical or interventional closure of the CF was recommended during childhood, even though they were asymptomatic, because of the risks that can occur during adulthood, including myocardial ischaemia, endocarditis and the complications of long-standing left-to-right shunt.^2^,^5^,^7^

Contrary to our case, nine of 10 CFs were asymptomatic in an evaluation of CFs in paediatric cases, and surgical ligation under CPB support without application of an aortic crossclamp was performed in four cases.^5^ According to Sakakibara et al.,^3^ our CF was type A in which ligation of the CF distal to the origin without CPB was recommended. Because the patient was symptomatic and due to the associated ASD, of which percutaneous closure was not feasible, we implemented ASD closure under CPB, suture closure of the atrial opening of the fistula from within the RA, and ligation of the CF outwardly, close to the connection with the RA.

## Conclusion

In our opinion, based on the clinical presentation and coexistent cardiac pathology, the choice of therapeutic management strategy should be individualised. Because of the possibility of the development of complications in the future, we successfully operated on a symptomatic paediatric case in whom the CF was incidentally diagnosed in association with an ASD.
